# Seroconversion of sheep experimentally infected with enzootic nasal tumor virus

**DOI:** 10.1186/s13104-015-1824-2

**Published:** 2016-01-07

**Authors:** Scott R. Walsh, Kevin J. Stinson, Sarah K. Wootton

**Affiliations:** Department of Pathobiology, Ontario Veterinary College, University of Guelph, Guelph, ON Canada

**Keywords:** Enzootic nasal tumor virus, Seroconversion, Virus neutralization

## Abstract

**Background:**

Enzootic nasal tumor virus (ENTV-1) is an exogenous betaretrovirus of sheep that transforms epithelial cells lining the ethmoid turbinates leading to a disease called enzootic nasal adenocarcinoma (ENA). A unique feature of ENA is the apparent absence of a specific humoral immune response to the virus, despite the highly productive infection in nasal tumors. The sheep genome contains approximately 27 copies of endogenous ovine betaretroviral sequences (enJSRVs) and expression of enJSRVs in the ovine placenta and uterine endometrium throughout gestation is thought to induce immunological tolerance to exogenous ovine betaretroviruses, a factor that may influence the likelihood of exogenous ENTV infection and disease outcome. Nevertheless, we recently demonstrated the presence of neutralizing antibodies directed against the ENTV-1 envelope glycoprotein in sheep naturally exposed to ENTV-1.

**Findings:**

Here, we employed an ENTV-1 envelope glycoprotein surface subunit specific ELISA and a virus neutralization assay to monitor serum antibody responses to ENTV-1 in a group of lambs experimentally infected with ENTV-1 virus containing filtered ENA tumor homogenate. Seroconversion and development of neutralizing antibodies was detected in one of six experimentally infected lambs.

**Conclusions:**

Our results demonstrate that sheep can respond immunologically and seroconvert following ENTV-1 infection suggesting that anti-viral immune responses may play a role in the development of ENA.

## Findings

Enzootic nasal adenocarcinoma (ENA) is an infectious neoplasm of the nasal mucosa of small ruminants [1]. Although often misdiagnosed, ENA is a common disease among North American sheep flocks, with a prevalence of up to 16 % in some Canadian flocks [2, 3]. Enzootic nasal tumor virus (ENTV), a betaretrovirus of sheep (ENTV-1) and goats (ENTV-2), has been implicated in the etiology of ENA [4–6]. We recently conducted experimental infections in newborn lambs demonstrating the transmission of ENA using cell-free tumor homogenate [6]; thereby verifying ENTV as the causative agent of ENA. Subsequently we developed a RT-PCR test for ante mortem detection and diagnosis of ENTV infection [3]. In that study, neutralizing antibodies reactive against the ENTV-1 envelope glycoprotein were detected in the serum of sheep from a flock with a high prevalence of ENA. However, correlation of antibodies with the presence of ENA lesions was only moderate, thus no further conclusions could be drawn as the exposure status of the sheep could not be conclusively determined.

The sheep genome harbors at least 27 endogenous betaretroviral sequences (called enJSRVs) that are highly related to the exogenous and pathogenic JSRV and ENTV sequences (90 % nucleotide sequence identity across the genome) [7]. EnJSRV transcripts can be detected in the thymus of fetal sheep [8], in a region of the thymus where the final selection of T cells occurs [9], as well as in Peyer’s patches. Consequently, sheep are thought to be immune tolerant to exogenous ENTV-1 via central and peripheral tolerance driven by enJSRV sequences. Indeed, earlier studies were unable to detect ENTV-1 capsid reactive antibodies in the serum of sheep with naturally acquired ENA [10]. In the present study, we endeavored to test whether sheep experimentally infected with ENTV-1 would seroconvert and develop antibodies against the ENTV-1 envelope protein.

The University of Guelph Animal Care Committee approved all animal use and related procedures. Six 2-day-old lambs (Rideau Arcott x Polled Dorset cross) from the University of Guelph specific pathogen free (SPF) flock, housed in isolation, were infected with filtered, cell free ENA tumor homogenate containing ENTV-1 via nebulization as previously described [6]. Blood samples were taken biweekly by venipucture of the jugular vein using serum-separating vacutainer tubes (Becton–Dickinson, Mississauga, Ontario, Canada). Serum samples were stored at -80 °C. Detection of antibodies reactive against the ENTV-1 envelope protein was performed using an indirect ELISA that was previously developed in our lab [3]. The antigen used in this ELISA, ESU-IgG, is a chimeric protein comprised of the surface (SU) subunit of the ENTV-1 envelope protein fused to the human IgG constant region [10]. Briefly, flat-bottomed 96-well plates (VWR International, Mississauga, Ontario, Canada) coated with purified ESU-IgG (2 μg/ml) were probed with heat inactivated (56 °C for 30 min) serum samples diluted 1:50 in blocking buffer. Horseradish peroxidase (HRP) conjugated rabbit anti-sheep IgG (Life Technologies, Burlington, Ontario, Canada) secondary antibody was used to detect binding of antibodies in the serum sample in conjunction with ABTS substrate [2,2′-azino-bis(3-ethylbenzthiazoline-6-sulfonic acid); Mandel Scientific, Guelph, Ontario, Canada] for color development.

**Fig. 1:**
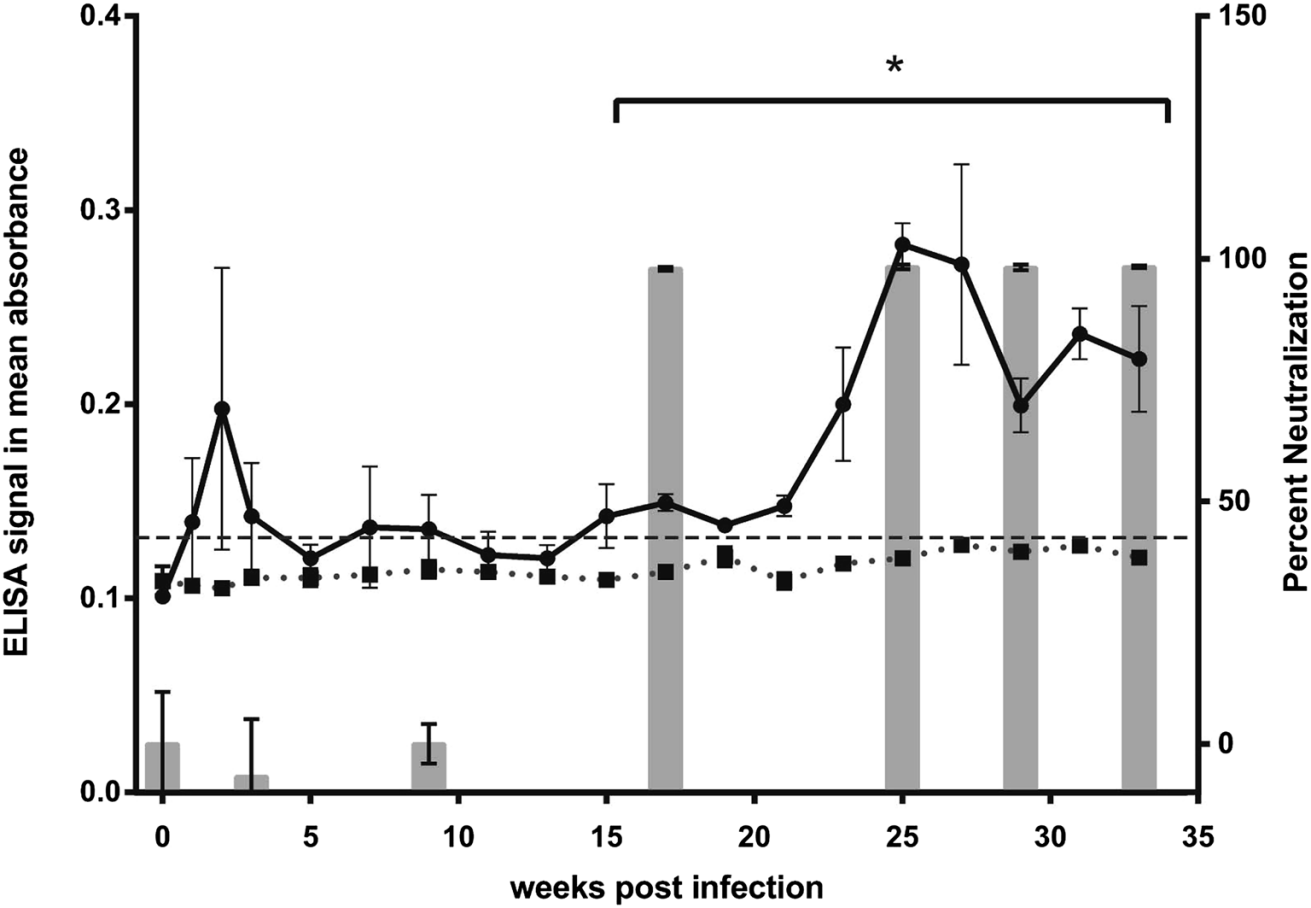
ELISA detection of serum antibodies reactive against the ENTV-1 envelope protein and causing neutralization of an ENTV-1 envelope protein pseudotyped retroviral vector. Sheep serum samples were evaluated in an indirect ELISA using purified ENTV-1 envelope (ESU-IgG) protein as the target antigen. The dashed line represents the background cut off value generated using serum from SPF sheep. The solid line with circular points represents results from the sheep that seroconverted following ENTV-1 infection and the dotted line with square points is representative of a non-seroconverter. ENTV-1 envelope pseudotyped MLV particles carrying the human placental alkaline phosphatase reporter gene were incubated with the indicated serum samples prior to infection of NIH 3T3/LL2SN cells [11]. The percent reduction in hPLAP positive foci is graphed

ELISA results are expressed as absorbance and are shown as a line graph in reference to the left axis in Fig. 1. Naive serum samples from an SPF research flock with no history of ENA (previously described [6]) were used to determine the background cut-off value (dashed line in Fig. 1), which was calculated as the mean of the naïve samples plus three times the standard deviation of those samples. Antibodies reactive against the envelope protein of ENTV-1 were detected in the serum of one of six infected sheep (solid line and circular points in Fig. 1). In this animal, envelope specific antibodies first appeared two weeks post-infection, but absorbance values were not significantly higher than the cut-off value. Serum from this animal remained similar to the cut-off value until 23 weeks post-infection at which point the absorbance values rose slightly, became significant, and maintained significance for the remainder of the study. The remaining five experimentally infected sheep displayed no evidence of seroconversion at any time point when assayed using the ESU-IgG ELISA. Representative absorbance readings for one these non-seroconverters are shown as a dotted line with square points in Fig. 1. We next tested the serum samples in a virus neutralization assay to verify the seroconversion observed in the ELISA and to determine whether this antibody response was neutralizing. Serum neutralization of a murine leukemia virus (MLV) pseudotyped with a C-terminally truncated version of the ENTV-1 envelope protein (Eenv I575*) and expressing a heat stable human placental alkaline phosphatase (hPLAP) reporter gene was performed as described previously [6]. Serum neutralization results are expressed as a percent reduction in hPLAP positive foci after serum treatment and are shown as grey bars in reference to the right axis in Fig. 1. Serum samples obtained prior to experimental infection as well as three and nine weeks post-infection did not significantly reduce the number of AP positive foci. However, a dramatic reduction in AP positive foci (>90 % reduction relative to untreated virus) was observed for serum samples from 17, 23, 25 and 28 weeks post-infection. The profound reduction observed at 17 weeks post-infection demonstrates the emergence of neutralizing antibodies, which correlates with seroconversion detected by the ESU-IgG ELISA.

At 35 weeks post-infection, animals were humanely euthanized and the nose was serial sectioned from caudal to posterior. Nose sections of all animals were unremarkable by gross inspection and tissue samples taken for formalin-fixation and immunohistochemical staining for ENTV envelope protein [2] were negative for ENTV-1 envelope expression (data not shown).

## Discussion

In this study, we have demonstrated seroconversion and development of neutralizing antibodies against ENTV-1 envelope protein in an experimentally infected sheep. These results show that sheep can respond immunologically to exogenous ENTV-1 envelope antigen. Seroconversion was rare; occurring in only one of six animals, but since none of these animals developed nasal tumors or had early neoplastic lesions that were detectable by immunohistochemical staining, we cannot ascertain whether the experimental infection was successful. Nevertheless, this data confirms our earlier finding that sheep can seroconvert and respond immunologically to ENTV-1 envelope protein [3]. The observation of two peaks in the ELISA results of the animal that seroconverted implies two distinct exposures to ENTV-1. The first peak occurs two weeks after experimental infection and correlates with the expected time frame for an antibody response generated due to experimental infection. However, the initially antibody response did not reach statistical significance and returned to baseline the following week. The brevity of the first peak suggests that the animal was initially infected, but there was a lack of persistent exposure of ENTV-1 envelope reactive B cells to the antigen. Conversely, the second rise in antibody response was robust and prolonged. It is likely that this peak was caused by the expansion and activation of a population of memory B cells, generated in the initial response, after a second exposure to the envelope antigen. The persistence of the second peak implies that the antigen exposure was also persistent. Early ENA lesions are known to express high levels of envelope protein [11] so release of virions from a developing ENA lesion could be the cause of memory B cell activation.

Previous studies have been unable to detect immune responses in sheep with ENA but these studies focused on antigens of the ENTV-1 capsid protein whereas our studies have shown sero-conversion to the envelope protein. When infected with ENTV-1, sheep initially did not mount a significant immune response, but did develop neutralizing antibodies against the virus after a lengthy incubation period. Neutralizing antibodies are an important factor in limiting spread of virus within the host. Therefore, the detection of neutralization antibodies specific to the ENTV-1 envelope protein in the serum of an experimentally infected sheep represents a biologically active antibody response to the virus infection. The envelope protein is a more biologically relevant target of antibodies during an ENTV-1 infection because the envelope is exposed on the virion surface whereas the capsid is hidden in the viral core. Therefore antibodies binding the envelope protein can potentially neutralize ENTV-1 infection and inhibit virus spread while capsid specific antibodies would be unable to bind and neutralize ENTV-1 virions. Additionally, neutralizing antibodies against the JSRV envelope protein have been detected in sheep experimentally infected with recombinant JSRV [12], another oncogenic sheep betaretrovirus that is closely related to ENTV-1 [13] and is similarly influenced by enJSRV sequence induced immune tolerance [8]. The prevalence of sero-conversion in this study (1 of 3 infected lambs) [12] was similar to what we observed.

The observation that antibodies can be detected in sheep naturally infected with ENTV-1 [3] does not conflict with a possible enJSRV-induced tolerance. All processes involving tolerance, be it central or pheripheral, are recurring events and may be broken [14]. Several reports in the literature demonstrate that tolerance can be broken when self-antigens are detected, especially in large amounts, in the presence of pro-inflammatory signals (e.g. adjuvants) that promote the maturation of antigen-presenting cells [14, 15].

Taken together, these results demonstrate that sheep can respond immunologically to ENTV-1 infection and suggest that the role of the immune system in determining the outcome of ENTV-1 infection should be explored further. In addition, immunization strategies targeting envelope protein antigens may be a viable strategy for controlling ENTV-1 infection.

## Abbreviations

ENA: enzootic nasal adenocarcinoma
ENTV: enzootic nasal tumor virus
JSRV: Jaagsiekte sheep retrovirus
ABC: avidin-biotin-peroxidase complex
MLV: murine leukemia virus
hPLAP: human placental alkaline phosphatase
ELISA: Enzyme-linked immunosorbent assay
